# Trends in illegal wildlife trade: Analyzing personal baggage seizure data in the Pacific Northwest

**DOI:** 10.1371/journal.pone.0234197

**Published:** 2020-06-10

**Authors:** Rosemary T. Hitchens, April M. H. Blakeslee

**Affiliations:** 1 Department of Biology, Miami University, Oxford, Ohio, United States of America; 2 Department of Biology, East Carolina University, Greenville, North Carolina, United States of America; U.S. Geological Survey, UNITED STATES

## Abstract

The illegal import of wildlife and wildlife products is a growing concern, and the U.S. is one of the world’s leading countries in the consumption and transit of illegal wildlife and their derivatives. Yet, few U.S. studies have analyzed the illegal wildlife trade (IWT) on a national or local scale. Moreover, to our knowledge, no studies have specifically examined the trends associated with IWT moving through personal baggage. This work aimed to better understand the magnitude of illegal wildlife importation into U.S. ports of entry by determining trends associated with illegal wildlife products from personal baggage seizures, using the Pacific Northwest (PNW) as a specific case study. To identify the most influential factors determining the numbers and types of personal baggage seizures into PNW, we analyzed 1,731 records between 1999 and 2016 from the Fish and Wildlife Service’s (FWS) Law Enforcement Management Information System (LEMIS) database. We found five significant contributors: taxonomic classification of wildlife, categorical import date, wildlife product, source region, and the Convention on International Trade in Endangered Species of Wild Fauna and Flora (CITES) status. While wildlife seizures across taxonomic categories have generally decreased in the PNW since 2008, other findings provide a reason for concern. More specifically, mammals were identified as the largest animal group of seized wildlife, and temporal trends indicate increases in seizures for this and several other taxonomic groups. Many of the seizures originated from overseas, with East Asia serving as the largest source. Our PNW case study can be a model for how large-scale geographical seizure data can be used to inform about the major factors that have historically and presently contribute to IWT, with conservation implications globally.

## Introduction

Due to the rapid proliferation of the transnational illegal wildlife trade, the global community now considers it a serious crime to illegally import endangered species [1], with far-reaching consequences. Although the full extent of worldwide IWT is unclear, it is cited as one of the largest global black markets, generating lucrative monetary gains for those involved [2]. As the demand for endangered wildlife and their derivatives persist, suppliers use illicit methods and have facilitated a multitude of technological advances to expand the IWT black market [3].

The current dimension and growth of IWT pose a significant risk to global biodiversity, as well as to social and economic development [4]. IWT is a primary mechanism of global overexploitation and is driving a number of species to extinction [5]. The trade’s transnational nature also acts as an avenue for the spread of invasive species [6] and disease [7]. Lastly, social and economic concerns, such as the link between IWT and lost tourism revenue, have been raised [8]. These implications, among others, stress the importance of regulatory bodies and institutions to effectively monitor and restrict IWT [9].

To combat IWT, regulation has been implemented at international, national, and local levels. Each country has its own legislation regulating wildlife trade, but internationally, wildlife trade of parts and products (hereafter, all referred to as ‘wildlife’) is regulated under the Convention on International Trade in Endangered Species of Wild Flora and Fauna (CITES). Today, CITES has 181 signatory members and provides three levels of protection (Appendix I, II, and III) to over 35,000 species globally [10]. While scholars recognize the success of CITES as a platform for international cooperation [11], others note its limitations [12], particularly that species do not necessarily benefit from CITES without additional legislation and enforcement measures on a state and/or local level [13].

In the United States (U.S.), the FWS Office of Law Enforcement (FWS-OLE) is the primary authority for monitoring and intercepting wildlife trade regulated under CITES and domestic laws. Of the 328 U.S. ports of entry, eighteen are designated for wildlife shipments [14] and are staffed by a mere 130 inspectors spread throughout the country. It is commonly accepted that FWS enforcement officials are burdened by an overwhelming workload and thwarted by inadequate budget and staffing. This is especially noteworthy considering the U.S. is a key player in the IWT [15].

In fact, the U.S. is one of the largest consumer and transit countries of IWT worldwide, with an estimated market value of $2 billion per year [16], demonstrating a reasonable cause for concern [17]. Despite this, little is known about the scale and mechanisms of IWT in the U.S. [18]. A handful of studies have analyzed illegal wildlife in the U.S. [19–27]; yet the bulk of this literature examines broader international trafficking patterns [28–29] or focuses on a specific taxonomic group [30–34] and tend to be descriptive in nature, with a few exceptions [35–36].

To appropriately address wildlife crime on all levels, research needs to focus on remaining gaps in the literature, which are largely attributed to incomplete and unreliable data, as well as the difficult nature of studying the clandestine trade [37]. Nevertheless, seizure records obtained by law enforcement bodies can provide readily available data that may be used to evaluate the scale of IWT. Seizure records primarily come from the CITES database [38–40], the TRAFFIC database [41], the EU-TWIX database [42] or the U.S. FWS’s Law Enforcement Management Information System (LEMIS) database [43–50]. Using these databases, researchers can investigate illegal patterns and trade routes over a period of time and determine the major factors that drive IWT [51–52].

To our knowledge, no scholarly work has yet examined illegally transported species that are moved through personal baggage. Personal baggage seizures are a growing global concern as international travel has increased with time [53], with concomitant increases in the transfers of illegal wildlife through the air transport sector [54]. Moreover, there is often a general lack of knowledge surrounding the illegal import of living wildlife or wildlife derivatives moving internationally within a traveller’s personal baggage [55]. Understanding the factors that contribute to the transfer of wildlife derivatives in personal baggage can help reveal the most vulnerable wildlife groups, the types of wildlife products being moved, where the products are coming from, the conservation status of the illegally imported wildlife, as well as changing trends through time.

Here, we present a case study of personal baggage seizures in the growing Pacific Northwest (PNW) region of North America. Together with the Port of Tacoma, the Port of Seattle is the fourth largest container gateway in North America [56] and is home to the largest airport in the PNW, Seattle-Tacoma International (Sea-Tac), which ranks among the fastest growing airports worldwide [57]. Our study was borne out of a desire to understand IWT’s extent and scale in such a major U.S. shipping region, thus providing wildlife crime law enforcement a greater understanding of the what, why, when, and where of IWT in the PNW region, especially as situational crime becomes a popular topic in the literature [58]. Our study also adds to the growing understanding of worldwide IWT by analyzing large-scale geographical seizure data using a highly important global port as our model system.

### Theoretical framework

When holistically addressing illicit wildlife seizures in passenger baggage, we consider both consumer demand for wildlife and wildlife parts as well as the environment enabling wildlife crime. This approach follows previous efforts to combine cultural and green criminologies in an attempt to interpret the underlying demand embedded in illegal trade of wildlife [59] while still accounting for opportunities enabling wildlife crime. Wildlife species are illegally traded for various reasons, and it is largely understood that demand is driven by social and cultural reasons. For example, animal skins and furs are used for clothing and accessories [60,61], fish are traded for food delicacies, like caviar [62], small mammals, reptiles, and birds are removed from their wild environment for the pet trade [63–65], tiger bone and rhino horn are harvested for traditional Asian medicine (TAM) [66], and elephant ivory is sought after as a status symbol [67]. Alongside socio-cultural demand patterns, IWT is influenced by opportunity structures in the environment. A growing number of studies by criminologists examine IWT through the lens of environmental criminology [68–72]. (For a comprehensive overview of applying environmental criminology to IWT see Kurland and Pires [73] and Boratto and Gibbs [74]). While multiple disciplines lend theoretical models to help explain IWT, environmental criminology entered the landscape to further understand the crime event itself, focusing more on situational influences and less on the dispositional factors influencing individual actors to engage in illicit behaviour. According to the opportunity framework put forth by criminologists, declines in crime follow when opportunities to commit such types of crime are reduced [75]. In the context of IWT entering the PNW through personal baggage, the opportunity framework would expect concentrations as they relate to the exporting country, types of wildlife products, and groupings of taxonomic groups, among other variables. Once these concentrations are identified, focused response strategies deriving from situational crime prevention (SCP) techniques and our understanding of socio-cultural dynamics can be used to devise interventions and curb demand. In our study, environmental crime is used as a multifaceted framework [76] rather than a rigorous theoretical model [77] and as such, we draw on both opportunity theory and socio-cultural explanations when analysing our findings and formulating our policy recommendations.

## Methods

This study includes a 17-year (1999–2016) analysis of seized wildlife illegally entering four PNW ports: Seattle (Washington), Bellingham (Washington), Portland (Oregon), and Sumas (Washington). Data were acquired from the LEMIS database, through a formal Freedom of Information request made on August 11, 2016, which included all available refused, seized items in personal accompanying baggage at these PNW port (in the LEMIS dataset, ‘refused’ is the action, ‘seized’ is the disposition, and ‘personal accompanying baggage’ is the transportation code). U.S. seizure data show that most enforcement activity pertaining to IWT takes place at ports of entry, rather than at domestic entrances [78], providing a strong rationale for collecting data from these four PNW ports. We received 1,731 seizure records of metazoan animals dating back to January 1999, distinguished by port of import, country of origin, country of import/export, intended purpose (e.g., personal, educational), source (e.g., wild specimens, animals bred in captivity), wildlife product (e.g., whole dead animal, ivory jewelry), estimated value, size of shipment by quantity of individuals, generic name, and taxonomic classifications, including genus, species, and subspecies.

From the 1,731 incidents recorded in the original database, a duplicate database was created, and 593 records were removed by combining shipments that were discovered to be from the same seizure, which likely occurred when one seizure contained multiple species. These duplicate entries were determined as those where the shipment data, country of import, port of entry and taxa were all the same. After merging duplicates, we categorized variables into new groupings to produce reasonably sized variables for comparison purposes. First, countries of export (*Country IE*) were grouped into their respective geographic region (e.g., Asia) to create the variable *Region*. Second, dates (mm/dd/yyyy) were grouped into three-year timeframes (*1999–2001*; *2002–2004*; *2005–2007*; *2008–2010*; *2011–2013*; *2014–2016*) in order to create adequately sized categorical groupings to assess temporal effects of IWT into PNW. Third, our dataset was classified into ninety-four wildlife descriptions (e.g. baleen, feather, horn carving) that we placed into fourteen broader *Wildlife Products* categories (*Bone; Body/Parts; Coral/Shell; Ivory; Live; Leather; Food; Medicine; Feather; Jewelry; Clothing; Other; Horn; Rug;* S1 Table).

Lastly, more specific taxonomic categories (e.g., genus, species) were appropriately grouped into larger taxonomic categories to provide an understanding of the major types of animals involved in IWT. These primarily represented taxonomic classification at the Phyla and Class levels (*Actinopterygii*, *Artiodactyla*, *Carnivora*, *Cnidaria*, *Mollusca*, *Reptilia*, *Aves*, and *Other Mammal*), but they were not entirely consistent in ranking because some animal groups (e.g., mammals) are much better studied/understood at lower taxonomic levels than are other groups (e.g., cnidarians); moreover, illegal trade is not consistent with taxonomic level, and some groups are more likely to be included in IWT than others. These became our decided approaches to create the most meaningful data for analyzing influential factors of IWT seizures in the PNW region. For the purposes of this study, we treated each entry row as a unique incident while independently considering seizure quantities (i.e., number of individuals per seizure) when appropriate. We also note here that we could only analyze whole number counts, not weight, as it is difficult to compare weights of different species (e.g. the weight of 660 seahorses cannot be appropriately compared to the weight of one grevy’s zebra).

We further analyzed data in terms of species type and protected status by manually adding information pertaining to species protection and taxonomic categorization into the LEMIS derived database. Species protection was obtained from both CITES and the International Union for Conservation of Nature (IUCN). The IUCN *Red List* [79] assesses the extinction risk of threatened species and classifies them into one of three categories: vulnerable, endangered, or critically endangered. CITES regulates the wildlife trade by assigning species into one of three Appendices: I, II, and III. If identifiable, each seizure was also classified as marine or terrestrial. In addition, after grouping wildlife descriptions into the fourteen product categories, they were designated as either dead or alive (97% of seized wildlife were dead–often transformed into a product; S1 Table). It should be noted that 563 (32.5%) wildlife specimens and products in the database were not identified to species level, likely owing to inherent difficulties in distinguishing an animal or product to species level for reasons such as sample degradation, unfamiliar taxa, transformation into a product, etc.

### Statistical analyses

To identify the most significant factors driving IWT seizures in the PNW, we fit a negative binomial (log link) generalized linear mixed model (GLMM) to the data with quantity of seized animals (counts) as our response variable, port code as a random effect, and the following parameters as fixed effects: CITES status, IUCN status, Marine/Terrestrial, taxonomic Class, Wildlife Product grouping, Region of origin, and Categorical Date (S2 Table). Since the database included a number of possible independent variables that could influence the quantity of seized wildlife in personal baggage, we chose to analyse the data using information criteria. This approach evaluates a number of possible models to determine the model(s) (and their included factors) that best fit the data. We therefore evaluated an exhaustive suite of models using the parameters and statistical methodologies described above, and then compared each model to one another using the corrected Akaike’s Information Criterion (AICc). The model possessing the lowest AICc value is identified as the best performing model. However, other models may be statistically as likely (or close to likely) as the model with the lowest AICc, making these additional models also potential candidates for the data. To determine if we had multiple high performing models, we first determined the model with the lowest AICc and then calculated each model’s ΔAICc value and corresponding AICc weight (w_i_). This provided a measure of model likelihood normalized by the sum of all model likelihoods. Models with higher model weight values represent greater proportional likelihood than those with lower model weights, thereby providing a measure of the strength of the best fitted model against all other evaluated models. This information criteria approach has been utilized in a number of published analyses across multiple biological disciplines, including conservation related topics [e.g., 80–82]. Of the evaluated parameters, five (IUCN, Class, Products, Region, and Date) were revealed as significant fixed effects (see Results). These were expanded upon using descriptive statistics and visual aides to determine relationships between and among variables, when appropriate. Data analysis were performed using JMP Pro v14.0.

## Results

Between January 9, 1999 and September 29, 2016, a total of 1,731 seizures were recorded, involving a total of 583,735 individuals from 4 ports in the PNW. Over this 17-year period, the total number of imported wildlife shipments denied clearance each year ranged from 27 in 1999 to 179 in 2006, with an annual average of 96 seizures. Seized wildlife at all four PNW ports originated from a total of 72 countries.

**Fig 1:**
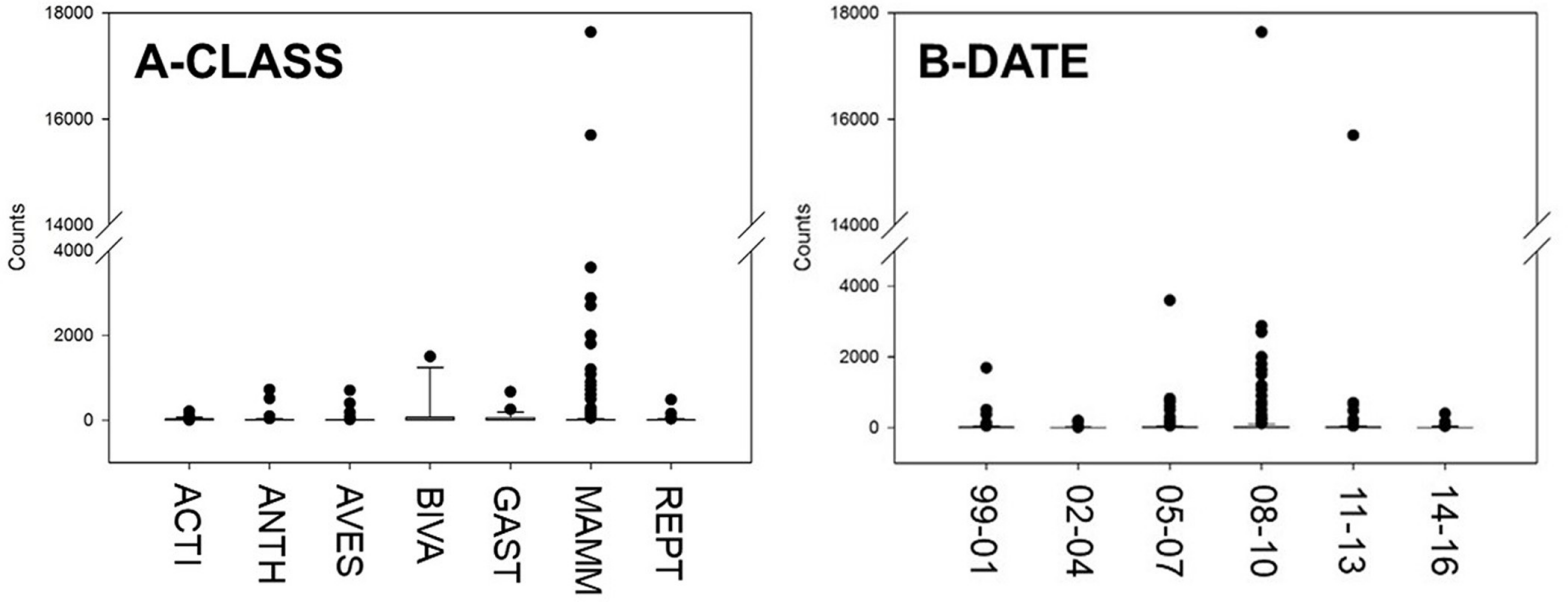
Factors identified as influential to personal baggage wildlife seizures in the PNW in the top model. Panel A demonstrates the taxonomic Class of organisms that were seized from personal baggage in the PNW (ACTI = *Actinopterygii* (Bony Fish), ANTH = *Anthozoa* (corals), AVES = *Aves* (birds), BIVA = *Bivalvia* (bivalves), GAST = *Gastropoda* (snails/slugs), MAMM = *Mammalia* (mammals), REPT = *Reptilia* (Reptiles). Panel B refers to the categorical date (3-year periods) of the seizures, including 1999–2001, 2002–2004, 2005–2007, 2008–2010, 2011–2013, and 2014–2016.

**Fig 2:**
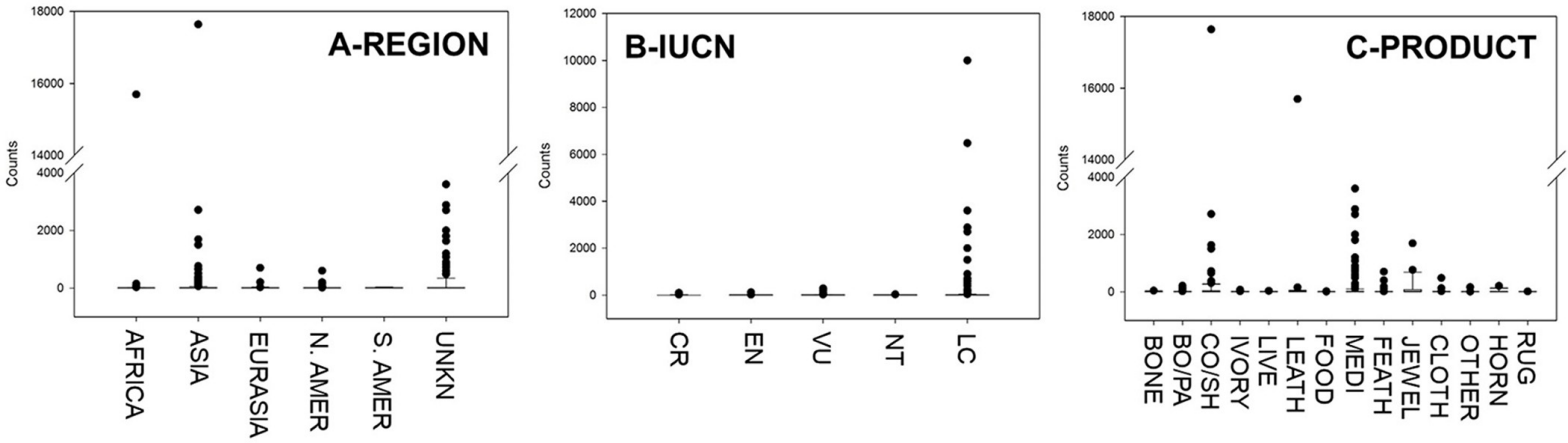
Three other factors identified as potentially influential in explaining patterns of personal baggage wildlife seizures in the PNW. Panel A represents the continent of origin into the PNW, including Africa, Asia, Eurasia, North America, South America. UNKN refers to unknown origin. Panel B demonstrates IUCN status (CR = critically endangered, EN = endangered, VU = vulnerable; NT = near threatened, and LC = least concern). Panel C demonstrates different wildlife product groupings among the seizures (BONE = bones and bone products; BO/PA = whole bodies and various body parts; CO/SH = coral and shells; IVORY = ivory products; LIVE = live organisms; LEATH = leather products; FOOD = food items; MEDI = medicinal items; FEATH = feathers; JEWEL = jewelry; CLOTH = clothing; OTHER = miscellaneous items; HORN = whole horns or horn products; RUG = items made into a rug).

Our GLMMs generated a number of models with extremely low AICc weights, and only two models had AICc weights >0.01 (Table 1), suggesting strong support particularly for the top model. The best fit model had a model weight of 0.981 and included two parameters: Class (F = 2.407; p = 0.011) and Categorical Date (F = 0.419; p = 0.836) (Fig 1). The second model had a weight of 0.011 and included three parameters: Class (F = 2.310; p = 0.014), Categorical Date (F = 0.199; p = 0.963), and Region (F = 0.247; p = 0.941). The third and fourth models had low model weights of 0.004 and 0.002, respectively. These two models included Class, but additionally two new parameters, IUCN status and Wildlife Product grouping (Table 1, Fig 2).

**Table 1:** Information theory approach for determining best fit models of illegal seizures of wildlife (counts) into PNW.

Model	Parameters	# Para-meters	AICc	ΔAICc	w_i_
1	Class, Date	2	17713	0	0.981
2	Class, Date, Region	3	17722	9	0.011
3	IUCN, Class, Product	3	17724	11	0.004
4	IUCN, Class, Region	3	17725	12	0.002

Each model represents a negative binomial mixed model with fixed parameters of CITES status, IUCN status, Marine/Terrestrial, taxonomic Class, Wildlife Product grouping, Region of origin, and Categorical Date (see Table 1), and Port Code as a random effect. F = F statistic; P = p value; AICc = corrected AIC; ΔAICc = subtracts the lowest AICc value from the AICc value being assessed; w_i_ = refers to the model weight as a proportion of all possible model weights. The table represents all models with model weights greater than 0.002.

Taxonomic grouping (Class; Fig 1A) and temporal investigations of IWT (Fig 1B) revealed that while trade in wildlife is still occurring in the PNW, overall seizures have decreased over time since their highest peak in the mid-2000s (Fig 2). This finding also highlights how seizures across many of the taxonomic groups (excluding Actinopterygii) increased to their highest level in the 2005–2007 time period and have steadily been decreasing since the 2008–2010 period.

In addition to temporal and taxonomic classifications, the second model also included Region as an influential factor (Fig 2A). In our dataset, we grouped 72 countries into larger regional categories (S2 Fig), finding East Asia (46%) to be the region from which the greatest proportion of personal baggage had been seized for illegal wildlife products, followed by North America (22%), Asia (18%), Africa (9%), Eurasia (3%) and lastly South America (1%) (S1 Fig). In addition, 19% of seizures to PNW were of unknown origin.

While models one and two did not consider CITES or not IUCN status as a significant factor, the third and fourth models included IUCN status (Fig 2B). When examined more closely, we found that the majority (60%) of total seizures by IUCN Red List status were organisms classified as Least Concern, and that the remainder were identified as Vulnerable (26%), Endangered (5%), Near Threatened (5%), and Critically Endangered (4%) (S2 Fig).

Finally, the third model also considered Wildlife Product grouping as a significant parameter (Fig 2C). After collapsing the types of wildlife products into broader groups, we found that illegal wildlife products related to medicinal use, coral/shell, and leather had the highest mean counts across all those seized in personal baggage during the 17-year period.

## Discussion

Our results suggest that a disproportionate share of taxonomic groups, time, export regions, wildlife product, and species at-risk status account for a majority of the incidents in our dataset, as is expected under an opportunity-based framework. Digging further into these trends using socio-cultural research will help us explain why such patterns occur among the significant variables analysed in our dataset. Below, we discuss our major findings using the PNW as a case study and then put our results into the larger context of IWT around the world.

### PNW personal baggage seizures over time

**Fig 3:**
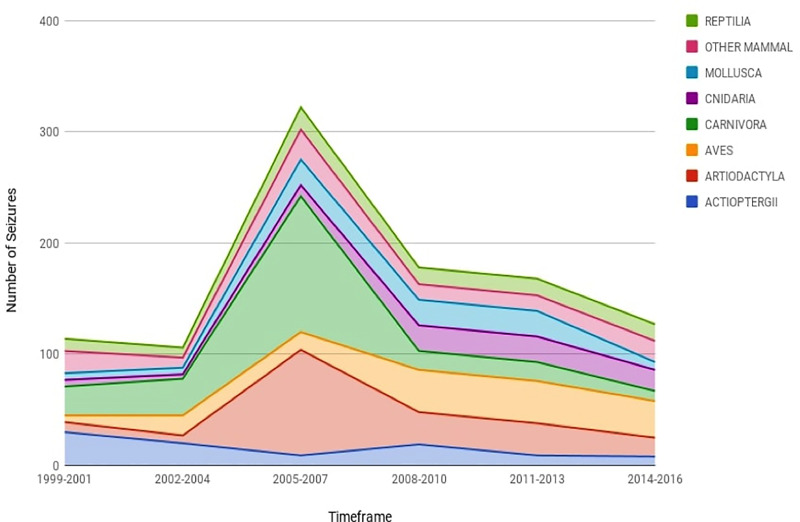
Seizures in the PNW by taxa across time. The figure includes the number of seizures in the PNW by taxonomic classes analyzed throughout the study period (1999–2016).

Although illegal trade in wildlife is still occurring in the PNW, overall personal baggage seizures across most taxonomic groups (excluding *Actinopterygii*) have steadily been decreasing since the 2008–2010 time period after reaching their highest peak between 2005–2007 (Figs 1A–1E, 2 and 3). Despite the fact that wildlife trafficking involves distinct markets, each with their own dynamics, the data suggest that regardless of species or taxonomic group, wildlife seizures across multiple taxonomic groups in the PNW may collectively be affected by particular events or circumstances through time. We suggest three possible factors that may help explain the decline in personal baggage seizures in the PNW since the mid-2000s. First and seemingly most likely, is the elimination of an international mail facility located at Sea-Tac International Airport prior to 2010. Before its removal, this mail facility presumably received a high volume of wildlife shipments, some of which could have been documented as personal baggage. For example, inspection blitzes in 2005 and 2009 examined over 500 shipments, which resulted in six seizures of the succulent, *Hoodia*, as well as CITES Appendix II butterflies, and coral and shell jewelry [83]. Second, illegal wildlife products in personal baggage could be slipping through inspections undetected, but according to the Country Enforcement Index, the U.S. successfully detects and seizes illegal wildlife products traveling through its airports 84.6% of the time [84]. This high number indicates effective enforcement among FWS-OLE officers. The third possible reason for the decline in personal baggage seizures through time is that the PNW trends reflect national or global trends, such as lower demand for certain products or taxa groups. According to FWS-OLE officers in the PNW, the quality of TAM has altered, driving different specimens and their products through personal baggage. It remains unclear which of these, or other factors influenced the decrease in seizures in the PNW in the late 2000s; and moreover, whether the peak in seizures between 2005–2007 is only high relative to the retrospective drop with the elimination of international mail facility; nevertheless, these observations raise interesting questions about the temporal fluctuations that can occur in IWT.

### PNW seizures by taxonomic class

Wildlife seized at the four PNW ports over the 17-year period belonged to five primary groups—mammals, birds, marine invertebrates, reptiles and bony fish (Table 2, Fig 1B). A study analyzing U.S. national seizures found relatively similar results: 94% of species seized at U.S. ports of entry between 2003 and 2013 belonged to six groups—mammals, molluscs, birds, reptiles, fish and coral—with mammals and reptiles making up more than half of seizures [85].

**Table 2:** Top 5 most frequent taxonomic classes by number of seizures.

Rank	Class	Number of Seizures	Percentage of Seizure
1	Mammalia	783	45.20%
2	Aves	265	15.20%
3	Anthozoa	160	9.24%
4	Reptilia	142	8.20%
5	Actinopterygii	106	6.12%

The data in this table include taxonomic classes and their ranks in terms of number of seizures and percentage (out of 100%) among all seizures.

The most noteworthy taxonomic group represented in the PNW seizure data were mammals, which showed the highest number of seized products at 450,446 in total. This finding is consistent with other seizure analyses that find mammal and mammal derivatives to constitute a majority of overall seizures [86]. As seizures in the PNW appear to be representative of IWT on a national level, this suggests that domestic trends are relevant and tractable. Of mammals, the family *Felidaes* accounted for the highest seizure count (n = 122), which included a large number of leopards (n = 93) and tigers (n = 14). Twelve of the 14 (86%) tiger seizures in our dataset were classified as medicinal products. Similarly, 92% of leopard seizures were categorized as *medicine* and the other 7 as *body/parts*. This finding aligns with the global trend of tiger skin declines from 2000–2015, replaced by an increase in seizures of tiger bones that are used in traditional medicines [87]. In the PNW, FWS wildlife inspectors have observed a surge in ‘higher-quality’ traditional Asian medicine products, including pills and plasters made with leopard products [88]. A study conducted by the WWF and World Conservation found that nearly half of their surveyed shops in seven North American Chinatowns, including those in Vancouver and Seattle, sold illegal wildlife products (note: Vancouver was not included in our study as we obtained data from the USFWS under LEMIS and did not pursue data from the Canadian government, but the close proximity of Vancouver to Seattle suggests similar trends could traverse these neighboring large cities) [89]. The sale and availability of TAM in Seattle and Vancouver is believed to be due to their high Asian populations, which are relatively greater than other cities in North America. In fact, 43% of Vancouver residents and 13% of Seattle residents have an Asian heritage [90]; this number increases as you consider the cities’ suburban populations, like Redmond, Bellevue, and Richmond [91].

Also in the group *Mammalia*, the *Moschidae* or Musk Deer, accounted for 20% of the seizures. The majority (94%) of the musk deer seizures originated from China and were categorized as *medicine*. This aligns with global musk deer trade routes, which tend to originate in Russia or Mongolia and are transported to China or other parts of East Asia for use in TAM. Concern for the illegal poaching of musk deer in Russia and Mongolia arose as early as the 1950s and prior research suggests a problem with enforcement measures and trade controls in these countries [92]. In addition, the *Ursidae* family made up 11% of mammals in our dataset, and seized bear products were primarily categorized as *body/parts* (25%) and *medicine* (51%). Bear paws and claws are considered a delicacy in Asia and bear gall bladders, as well as the bile that is derived from the gall bladders, are used in traditional medicine to treat a number of ailments [93]. Of the seized products belonging to the *Elephantidae* family, 45% were ivory products. In an effort to deter elephant ivory imports, which have been illegal by federal and international law for decades, West Coast states are passing their own laws. In the state of Washington, Initiative 1401 criminalized ivory sales throughout the state, and in Oregon, Ballot 100 effectively did the same. While an ivory seizure is not a common occurrence at PNW ports, it occasionally transpires, and the U.S. continues to remain a large ivory market [94]. Lastly mentioned is the *Phocidae* family, accounting for 6% of mammal seizures, which were largely made up of seal medicine products. This aligns with global trends of increasing seal derivatives in the TAM market, like a fur seal’s penis, which can be purchased from Beijing restaurants for USD $400 [95] due to its popularity as an aphrodisiac.

At 15%, *Aves* (birds) made up the second largest class in our dataset. While the illicit trade in birds is often attributed to the pet trade [96], the high seizure count of *Aves* in our case study is likely attributed to U.S. federal law, existing independently of international CITES protections. The Migratory Bird Treaty Act (MBTA) makes it illegal to import, export, or transport any migratory bird, or the parts, nests, or eggs of such a bird without a permit. When analysing our dataset, we found a number of migratory bird species, including but not limited to Red-Tailed Hawk (*B*. *jamaicensis)*, Bald Eagle (*H*. *leucocephalus)* and Great Blue Heron (*A*. *Herodias*). The most prevalent bird represented in our dataset was the Canada Goose (*B*. *Canadensis)*, a non-threatened species making up just over 10% of all *Aves* seizures in the form of feathers and meat. In all of these cases, the Canada Goose was imported from Canada, an important country for bird-related seizures, accounting for 84% of all *Aves* seizures. This is not particularly surprising considering the proximity of the PNW designated ports to the U.S.-Canada border. While our dataset did contain non-migratory birds and their derivatives, such as a seizure of 6,480 Indian peafowl (*P*. *cristatus)* feathers from China, these cases were few and far between. Another noteworthy finding is that 72% of *Aves* seizures in our dataset were feather products. These findings suggest that the MBTA is being enforced and upheld by enforcement officers in the PNW. Moreover, it’s possible that travellers are unknowingly trafficking migratory bird parts. Despite the fact that the MBTA is among the oldest wildlife protection laws in the U.S., there is little research on public perceptions of the act, which might change as the USFWS solicits public input and seeks to clarify the scope of the act [97].

The third largest class represented in our dataset, *Anthozoa* (marine invertebrates), make up just over 9%. Marine invertebrates (e.g., staghorn coral), alongside marine tropical fish, are in high-demand for U.S. home and public aquariums, driving a major global black market [98] that introduces foreign species to local communities and thus may contribute to the spread of invasive and nuisance species [99], along with a decline of these organisms in their source regions [100]. The U.S. is the world’s largest consumer and importer of coral reef associated species for ornamental purposes and Seattle has been identified as a primary point of entry for shipments of corals, coral products and reef associated species [101]. Of the *Anthozoa* seizures represented in our dataset, 80% were reported under the generic order *Scleractinia*, with few references to specific families or species within that order, a data deficiency also observed on a national level [102]. *Scleractinia* includes all stony corals, a group of hard skeleton marine animals that are under pressures as they seemingly prepare for a mass extinction, according to recent research [103]. Most of the coral seizures (77%) were classified as *coral/shell*, with a small handful of jewellery and live seizures. Live specimens comprised 14% of *Anthozoa* seizures, suggesting the role of the marine aquarium curio trade. The *Anthozoa* seizures in our dataset largely originated from the Philippines (61%) and were imported from the Philippines (54%), suggesting a strong trade route between the Philippines and PNW ports of entry in marine invertebrates.

The fourth largest taxonomic group represented in the PNW seizure data were reptiles at 8%. This contrasts with global trade analyses, which find reptile skins trafficking to be a relatively uncommon practice [104]. In the U.S., the illicit trade in live reptiles is largely attributed to their growing popularity as pets [105,106], which is highlighted by large-scale smuggling operations, one even reaching Washington state when a reptile smuggler was charged with importing more than 230 reptiles from Thailand [107]. Our findings differ from such patterns observed. Among PNW seizures, 95% of *Reptilia* specimens were dead, 32% of which were categorized as *leather* products and 31% as dead *body/parts*. Furthermore, while Latin America is a frequent source of reptiles destined for the U.S. [108], the majority of *Reptilia* seizures in this dataset originated from Asia (42%). On an international level, the heightened demand for reptiles is increasingly leading to overexploitation of species [109] and research suggests that reptile species, like crocodile skinks are overlooked by CITES [110]. The illicit trade of reptile skins often runs parallel to the legal trade, which is facilitated by the fashion industry in U.S. and European markets [111]. In the U.S., states are taking their own action to protect reptiles. California, which is believed to be 30% of the world’s alligator skin market [112], instituted a ban in alligator and crocodile products in 2019 in an effort to curb wildlife trafficking. While most California skins are produced from farmed and legally hunted alligators in southern U.S. states like Louisiana and Florida, it is believed that a portion of these products are derived from illegally caught species, as poaching activity suggests [113]. Our high seizure count of reptile body parts and products raises concern about an illicit reptile skin trade derived from Asia, and also raises questions about policy responses to a convoluted trade of legal/illegal reptile parts and products.

Lastly, *Actinopterygii* (bony fish) accounted for just over 6% of our dataset. As most all of the bony fish observed in our dataset were sturgeon, we attribute the high seizure count in bony fish to sturgeon international trade laws and enforcement efforts in the U.S. and Russia, as well as the development of a global aquaculture industry for sturgeon. Our data show that bony fish seizures were at their highest level in the late 1990s, which is likely due to the collapse of sturgeon management systems alongside the dissolution of the Soviet Union, leading to the large-scale criminalization of wild sturgeon stocks [114]. Moreover, in 2000, the OLE joined efforts with Russian authorities on caviar trade issues [115], and PNW wildlife inspectors focused their efforts on preventing illegal imports of caviar, resulting in the confiscation of 22 pounds of sturgeon caviar worth more than $16 million USD in 2001 [116]. Presumably, these targeted enforcement efforts led to the ongoing high level of *Actinopterygii* seizures in the PNW in the early 2000s. By the mid-2000s, sturgeon stocks had plummeted from overfishing and poaching in locations like the Caspian Sea, with the U.S. consuming over half the world’s export of beluga caviar [117]. The U.S. reacted by instituting a unilateral ban on beluga imports in 2005, and two years later, Russia implemented bans on sturgeon harvest [118]. These bans on sturgeon capture and import correspond with a decrease in PNW *Actinopterygi* seizures (Fig 2) and North American beluga imports [119].

Alongside regulatory and enforcement efforts, market changes also help explain declining *Actinopterygii* seizures in the PNW. As sturgeon populations dropped in the late 20^th^ century, an aquaculture industry for farmed sturgeon developed to meet demand, primarily for caviar [120]. With wild sources disappearing and becoming more difficult to obtain, farmed products gradually overtook the market [121]. The market shift from wild to captive bred sturgeon is likely another reason for the decrease in *Actinopterygii* seizures in the PNW. Adding farmed agriculture to the market makes illegal trade less lucrative, assuming that farming increases supply, thereby reducing prices and incentives to illegally capture wild stocks.

*Actinopterygi* seizures increased again in the PNW during the 2007–2010 period despite the fact that wild caviar supplies were declining [122]. One possible explanation for this increase in seizures amongst dwindling stocks and protectionary measures for wild sturgeon is the increase in illegal sturgeon exports, as North America became a supplier of caviar. This was highlighted in a FWS-OLE *FY 2008 Annual Report*, which detailed multiple cases of caviar dealers illegally exporting and selling shovelnose sturgeon in the U.S. [123]. The correlations between bony fish seizures in the PNW and such wildlife trade policies suggest that regulatory and enforcement measures do play a key role in the IWT for particular taxonomic groups that are prized as commodities.

### PNW seizures by geographic region of origin

In regional analyses, our findings support the commonly held notion that most seizures originate from East Asia (46%) and the remainder of Asia (18%) (S1 Fig). Similarly, a 2010 study by Rosen and Smith analysed 12 years of international seizure records, finding that most products were exported from Southeast Asia, also a hotspot of emerging infectious diseases [124]. Globally, Asia functions as a supplier for multiple aspects of IWT, including traditional medicine, the pet trade, food, and trophies [125]. In one case, a resident of Snohomish County, Washington was caught smuggling protected Asian reptile species, including but not limited to, Gila monsters, three-toed box turtles, and the critically endangered Arakan forest turtle, with an estimated value between $120,000 and $200,000 [126]. North America accounted for just under a quarter of the seizures, over 90% of which originated and were exported from Canada. Both Canada and the U.S. are large consumers and suppliers of IWT [127]. While Mexico is a top country for export of illegal wildlife shipments into the U.S. [128,129], our findings suggest that Canada dominates the trade of illegal wildlife into the PNW. This is likely due to the shared border between Canada and the state of Washington. Africa, on the other hand, accounted for 9% and Eurasia 3%, but this is likely because Europe is recognized as more of a consumer and transit region of IWT rather than an export region [130]. South America accounted for a mere 1% of PNW seizures and Central America less than 1%, which is surprising considering the high amount of IWT detected at other U.S. ports of entry for products like tortoiseshell bracelets [131]. It is also of note that 19% of the seizures were classified as having an unknown origin, which is a problematic classification because it cannot provide researchers, department personnel, and policy-makers with an understanding of where the products are coming from, likely leading to management uncertainty and inaction.

### PNW seizures by at-risk status

Regarding trends based on IUCN status, the majority of the seized species in the PNW were considered of “Least Concern” by the IUCN Red List (S2 Fig). In the U.S., regulation in wildlife is largely determined by CITES legislation and agreements with other signatory nations, but species are not necessarily protected or regulated if they appear on the IUCN Red List. Therefore, it is possible that wildlife are seized due to CITES protection measures rather than Red List status. Even so, this does not necessarily mean that those species classified as “Least Concern” are not endangered; in fact, evidence finds that the IUCN may be underestimating the number of species at risk of extinction [132] and the listing process can be lengthy and stalled by political decision-making [133].

### PNW seizures by wildlife product type

Lastly, wildlife products, specifically those related to medicinal use, coral/shell, and leather were found to be significant in our third and fourth model (Fig 2C), which suggests that certain types of wildlife parts or products (e.g. horn or ivory carvings) could be influential in driving ITW. As mentioned, traditional medicine is a primary driver of IWT across the world [134]. The curio trade is also an influential driver of IWT, which accounts for a variety of coral and shell parts and products, like red coral jewellery [135]. A *National Geographic* reporter found that just one facility in India processes as many as 100 tons of shells a month to meet demand for the curio trade, despite the fact that many of the shell species handled are protected under India’s Wildlife Protection Act [136]. Previous research finds that mollusc shells are one of the most heavily confiscated wildlife products at ports of entry across the U.S. [137]. Of increasing concern is the role tourism plays in the illicit trade of shells and coral to meet demand for souvenirs [138–140]. Lastly, leather products account for a high number of PNW seizures. Commonly seized leather products at U.S. ports of entry include leather boots, handbags, and wallets made from protected reptile and marine species, like sea turtles, caimans, crocodiles, lizards, and snakes [141].

### Data limitations

There is ample research to support the contention that wildlife seizure data provide a baseline understanding of the impact of IWT; however, such studies still remain plagued by discrepancies [142]. For one, using wildlife seizures as a measure of impact is complicated because it can be difficult to csompare among species. For example, a seizure in the LEMIS dataset containing 280,800 Kaluga sturgeon eggs is inherently different from a seizure of 1 Burmese python. Therefore, using variables such as quantity alone, makes it difficult to get a true picture of the scope of the issue. However, these data provide some level of quantitative understanding of IWT into the U.S. Secondly, this study, among others analyzing seizure data [143], do not account for the ecological impact of seizures. For example, the ecological impact of illegally imported mallard ducks (*Anas platyrhynchos*) from Canada might be less severe than seizures of Atlantic Puffins (*Fratercula arctica)* from Iceland. Thirdly, seizure data is dependent on the data source and only captures trafficking attempts that were unsuccessful. Accordingly, seizures can be evidence of effective enforcement rather than high volumes of IWT. Continued research related to these knowledge gaps are therefore needed.

### Conclusions and significance to global IWT

This study contributes to the growing body of IWT research that has identified and explained major trends and patterns of illegal wildlife imports into the U.S. Our case study in the PNW found that illegal wildlife is disproportionately coming in certain forms (i.e. product type), at-risk status, taxonomic groupings across time, and from particular export regions. These patterns are most likely a reflection of socio-cultural demands for certain products and opportunities to illicitly traffic. While overall IWT in the PNW region decreased over the time period analyzed, it is reasonable to assume that it continues to be a persistent threat for law enforcement and regulatory bodies to contend with, especially considering the current and projected growth of the PNW region.

The PNW region is currently experiencing a booming economy [144] and a rapidly growing population [145]. Moreover, it is a large U.S. seaport with notable affluence—all factors that have the ability to facilitate greater illegal wildlife imports and exports. To respond to the region’s growth, Sea-Tac is expanding its North Satellite and will open a new International Arrivals Facility in 2020, which will allow the facility to process 2,600 passengers per hour [146]. Sea-Tac currently offers a number of flights to Asia [147], which is a ‘hotspot’ region for IWT, as ours and other data have shown for international trade patterns [148]. In fact, it is expected that these international routes will continue to expand. This suggests that demand reduction practices are appropriate for curbing IWT among personal travellers in PNW ports, which complements current efforts by local FWS-OLE and the Port of Seattle to educate travellers departing and entering PNW ports.

While we do not know whether there is a general lack of awareness related to most personal baggage seizures, it is assumed that at least some portion of seizures are due to unknowing travellers bringing in souvenirs or trinkets purchased while abroad [149]. Globally, a lack of awareness is cited as one of the main reasons for the illegal trade in endangered species [150] and a WildAid sponsored survey found that only one in five Americans know anything about the illegal wildlife trafficking problem in the U.S. [151]. Furthermore, according to the UNODC [152], “while enforcement agents must seize non-compliant shipments, many of these incidents appear to be the result of ignorance or negligence, not criminal intent.” Therefore, a public awareness campaign targeted at unknowing travellers entering and exiting ports of entry could have an impact on decreasing the number of seizures.

The results of the PNW case study here can also provide a better understanding of how management may be targeted to address and combat the issue of IWT around the globe. In particular, the SCP framework (described in the Theoretical Framework section) can offer ways to better understand management and policy actions that could address IWT. The framework focuses on situational determinants that reduce the opportunity to commit wildlife crime and make it more difficult and less rewarding. This model focuses on ‘near’ situational causes of crime by understanding *how* crimes are committed, and not *why* someone commits a crime; however, this does not suggest that the *why* is of lesser importance. SCP could be a readily adaptable model for port enforcement agencies, such as Customs and the USFWS, and is likely to have considerable impact in the short-term, especially considering that changing cultural and consumer demands in other countries on a wide-scale might be outside the scope of U.S.-based enforcement agencies.

The SCP framework offers prevention techniques grouped into the following categories: increasing risk, increasing effort, reducing reward, reducing provocations, and removing excuses [153]. The policy recommendations suggested by Petrossian et al. in their 2016 analysis on seized illegal wildlife entering the U.S could be adapted to high-trafficked regions like the PNW [154]. First, the Seattle-Tacoma International Airport could increase the risk of detection by increasing the number of USFWS OLE trained officers. The number of officers that are enforcing wildlife laws and policies today across the U.S. has remained largely unchanged since the agency was created 30 years ago [155]. Second, agencies in the PNW could work collaboratively with popular wildlife trade origin countries, such as those in Asia, to encourage exist screens before illegal wildlife arrive in the U.S. For example, given the strong illicit trade route of *Scleractinia* corals between the PNW and the Philippines (as identified in this study), U.S. enforcement agencies could encourage exit screening at Manila Ninoy Aquino Internatioal Airport (MNL) and other high-traffic ports of entry in the Phillippines. Third, ports of entry could alert travellers of the risk associated with IWT by posting visual signs and aids at ports of entry indicating that the import of illegal wildlife is a crime and will be punishable.

While “top-down,” enforcement-led responses will continue to play an essential role in curbing IWT, they can overshadow the payoffs associated with multi-faceted interventions that engage the local community in solutions to curb illegal wildlife trade. As argued by [156], engaging and supporting local populations through community-based conservation (CBC) creates the “necessary backbone for enforcement by providing an enabling environment.” In an effort to stem the trade in illegal wildlife and wildlife parts, it is essential that intervention combine both “top-down” enforcement and community-engagement approaches as appropriate. The findings of this study raise a number of important questions that require further analysis; nevertheless, they will likely serve as a useful reference tool to local law enforcement and management bodies.

## Supporting information

S1 Table Wildlife product groupings. Seized wildlife products were classified into larger groupings that were then condensed into 14 broader categories.
(PDF)

S2 Table Description of parameters. The table below provides detailed information on the different parameters that were explored as part of this dataset.
(PDF)

S1 Fig Regions of origin. This figure depicts PNW seizures during the study period (1999–2016) by geographic origin, grouped into regional categories.
(TIF)

S2 Fig Total seizures by IUCN status. This figure shows total PNW seizures during the study period (1999–2016) by IUCN Red List status.
(TIFF)
