# Supplementary material for: Trends in illegal wildlife trade: Analyzing personal baggage seizure data in the Pacific Northwest
Source: PLoS One. 2020 Jun 10;15(6):e0234197. doi: 10.1371/journal.pone.0234197 (PMC7286499; doi:10.1371/journal.pone.0234197)
Supplement: S1 Table — Seized wildlife products were classified into larger groupings that were then condensed into 14 broader categories. (PDF) [file pone.0234197.s001.pdf]

S1 Table. Wildlife Product Groupings. Seized wildlife products were classified into larger groupings that were then condensed into 14 broader categories. BONE=bones and bone products; BODY/PARTS=whole bodies and various body parts; CORAL/SHELL=coral and shells; IVORY=ivory products; LIVE=live organisms; LEATH=leather products; FOOD=food items; MEDI=medicinal items; FEATH=feathers; JEWEL=jewelry; CLOTH=clothing; OTHER=miscellaneous items; HORN=whole horns or horn products; RUG=items made into a rug.

**PRODUCT CATEGORIES (ABRV)**	**ORIGINAL WILDLIFE PRODUCTS GROUPINGS**
BONE	BOC=Bone product or carving, BON=Bones (including jaws, but not skulls), BOP=Bone pieces (not manufactured)
BODY/PARTS	BOD=Dead animal (whole animal), CAP=Carapaces (raw or unworked), CLA=Claw (including talon), EAR=ear (except when part of the whole trophy), FOO=Foot, HAI=Hair, HAP=Hair product (including paint brushes, etc.), SKI=Skin (substantially whole, including tinga frames), SKU=Skull, SKP=Skin pieces (raw or tanned including scraps), SPE=Specimen (scientific or museum), TEE=Teeth (excluding tusk), TRO=Trophy (all the parts of one animal), TRI=Trim (shoe, garment, or decorative, TUS=Tusks (substantially whole tusks, worked or not), WNG=Wing
CORAL/SHELL	COR=Coral (raw or unworked, excluding live or rock coral), CPR=Coral products, ROC=Live Rock (e.g., Coral Rock), SHE=Shell (mollusc, raw or unworked), SPR=Shell product (mollusc or turtle)
IVORY	IJW=Ivory jewelry, IVC=Ivory carvings, IVP=Ivory pieces (not manufactured, includes scraps), PIV=Piano with ivory keys (# of pianos)
LIVE	LIV=Live specimens (live animals or plants)
LEATH	LPL=Leather product (large manufactured including briefcase, suitcase, furniture), LPS=Leather product (small manufactured including belt, wallet, watchband)
FOOD	CAV=Caviar (unfertilized dead processed sturgeon or paddlefish eggs), MEA=Meat
MEDI	MED=Medicinal part or product
FEATH	FEA=Feathers

JEWEL	JWL=Jewelry (other than ivory jewelry)
CLOTH	GAR=Garment (excluding shoe or trim), SHO=Shoe (including boots), TRI=Trim (shoe, garment, or decorative)
OTHER	CAR=Carvings (other than bone, horn or ivory), GAB=Gall bladders, GAL=Gall (bile), DER=Derivative (except those included elsewhere), MUS=Musk, UNS=Unspecified
HORN	HOC=Horn carving (including horn or antler products), HOP=Horn pieces (not manufactured), HOR=Horn (substantially whole including antlers)
RUG	PLA=Plates of fur skins (include rugs if made from several skins), RUG=Rugs (rugs if made from one skin only)
