# Supplementary material for: Trends in illegal wildlife trade: Analyzing personal baggage seizure data in the Pacific Northwest
Source: PLoS One. 2020 Jun 10;15(6):e0234197. doi: 10.1371/journal.pone.0234197 (PMC7286499; doi:10.1371/journal.pone.0234197)
Supplement: S2 Table — The table below provides detailed information on the different parameters that were explored as part of this dataset. (PDF) [file pone.0234197.s002.pdf]

TABLE 1. Description of Parameters

The table below provides detailed information on the different parameters that were explored as part of this dataset.

Parameter	Description
CITES	CITES protection status was obtained from the Checklist of CITES Species (www.checklist.cites.org), which provides the official list of CITES-listed species and the Appendix in which they are currently listed. Latin names were used (when provided) to manually look up each entry obtained from FWS. Appendix I lists species that are most endangered among CITES-listed animals and plants. Appendix II lists species that are not necessarily now threatened with extinction but that may become so unless trade is closely controlled. Appendix III is a list of species included at the request of a Party that already regulates trade in the species and that needs the cooperation of other countries to prevent unsustainable or illegal exploitation. For more details on the CITES Appendices, visit http://cites.org. For taxa higher than species level, seizures might have been identified as CITES I, II, or III, meaning species within the family are included in those Appendices. The last category assigned, "not protected" was assigned to all database listings not protected by CITES.
Class	The taxonomic Class for each seizure was included, when possible, in the LEMIS database.
Country of Export	The FWS-OLE LEMIS database includes over 245 countries that are coded for both country of origin and country of export. Wildlife inspection officers determine the country of export, when possible, usually based on accompanying paperwork.
Country of Origin	The FWS-OLE LEMIS database includes over 245 countries that are coded for both country of origin and country of export. Wildlife inspection officers determine the country of origin, when possible, usually

		based on accompanying paperwork.
	Date	Seizures received dated from 1999 to 2016. To create meaningful temporal data, dates (mm/dd/yyyy) were grouped into three-year timeframes: (1) 1999-2001; (2) 2002-2004); (3) 2005-2007; (4) 2008-2010; (5) 2011-2013; (6) 2014-2016.
	IUCN	IUCN protected status was obtained from the IUCN Red List of Threatened Species (www.iucnredlist.org/search), which provides taxonomic, conservation status, and distribution information on plants, fungi and animals that have been globally evaluated using the IUCN Red List Categories and Criteria. The lowest taxa possible were used to manually search for the FWS database entry using the IUCN Red List online database. The categories entered included: Critically Endangered (CR), Endangered (EN), Vulnerable (VU), Near Threatened (NT), Least Concern (LC), and Data Deficient (DD). A handful of seizures were not evaluated and did not receive IUCN categories. For more details on the IUCN assessment methods, visit http://iucnredlist.org.
	Marine/Terrestrial	If identifiable, each seizure was listed as marine or terrestrial. Entries were verified using The Catalogue of Life (www.catalogueoflife.com), external sources, or personal knowledge.
	Region	To create adequate numbers in all groups, the following countries were grouped into bigger regions: (1) East Asia: China, Japan, Taiwan, South Korea, Hong Kong and Macau; (2) Asia: All other Asian countries not included in East Asia, except for Turkey and the Middle East; (3) Eurasia: Europe, Russia, the former Soviet Republics, Turkey, and the Middle East; (4) North America: Canada, the U.S. and Mexico. Entries from South America and Australasia were removed because there were too few records.
	Transportation Code	This dataset only includes personal accompanying baggage. The following

		variables were filtered out and not included in our analyzes: air cargo, ocean cargo, rail, border crossing on foot, truck (or other commercial vehicle), mail, and personal vehicle.
	Wildlife Product	The FWS-OLE LEMIS database includes 95 wildlife descriptions (e.g. baleen, feather, horn carving) that we grouped into 14 wildlife product groupings: (1) Bone (2) Body/Parts (3) Coral/Shell (4) Ivory (5) Live (6) Leather (7) Food (8) Medicine (9) Feather (10) Jewelry (11) Clothing (12) Other (13) Horn and (14) Rug (see Table S1).
