# Supplementary figures and images for: Trends in illegal wildlife trade: Analyzing personal baggage seizure data in the Pacific Northwest
Source: PLoS One. 2020 Jun 10;15(6):e0234197. doi: 10.1371/journal.pone.0234197 (PMC7286499; doi:10.1371/journal.pone.0234197)

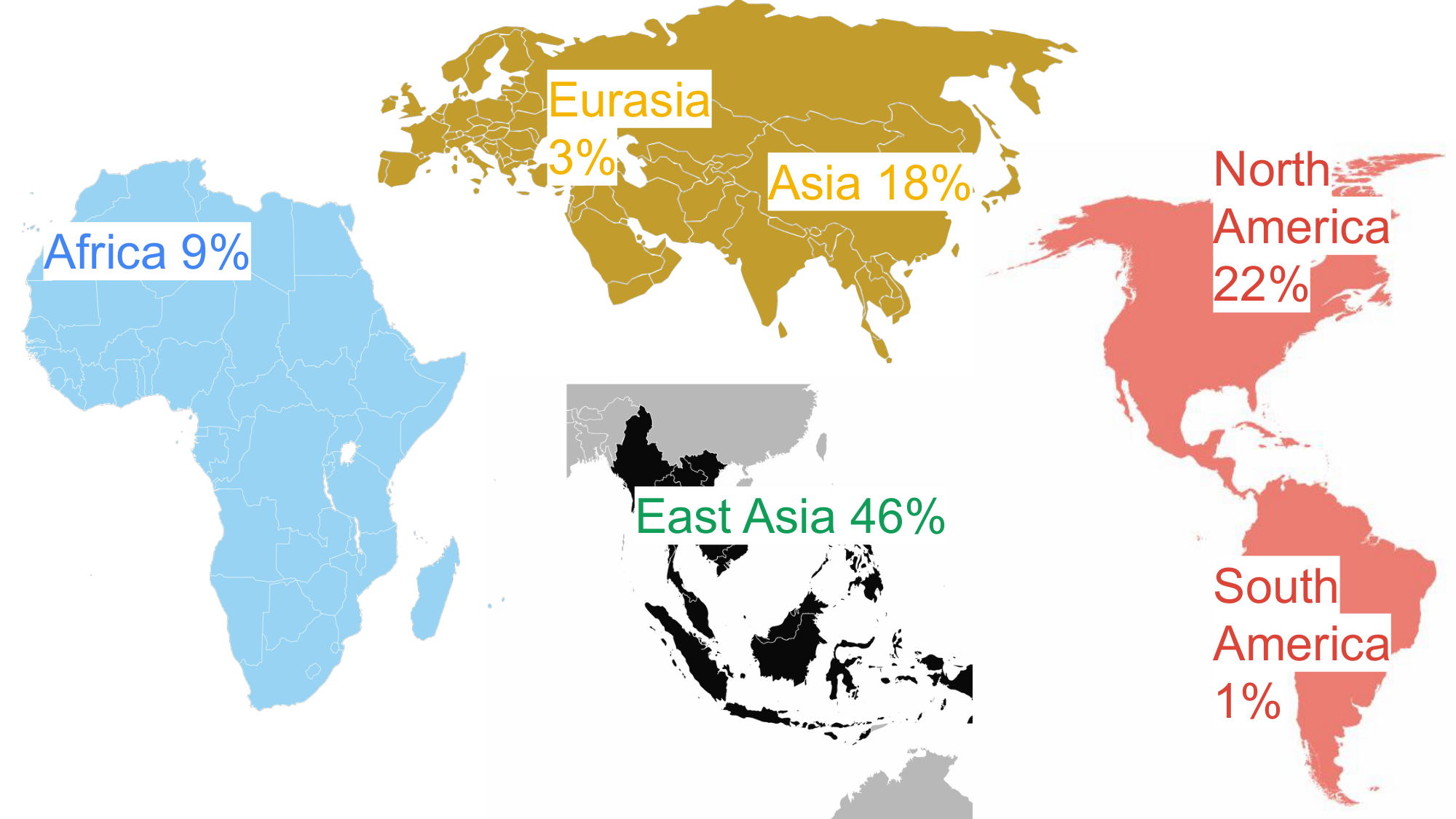

Supplement: S1 Fig — This figure depicts PNW seizures during the study period (1999–2016) by geographic origin, grouped into regional categories. (TIF) [file pone.0234197.s003.tif]

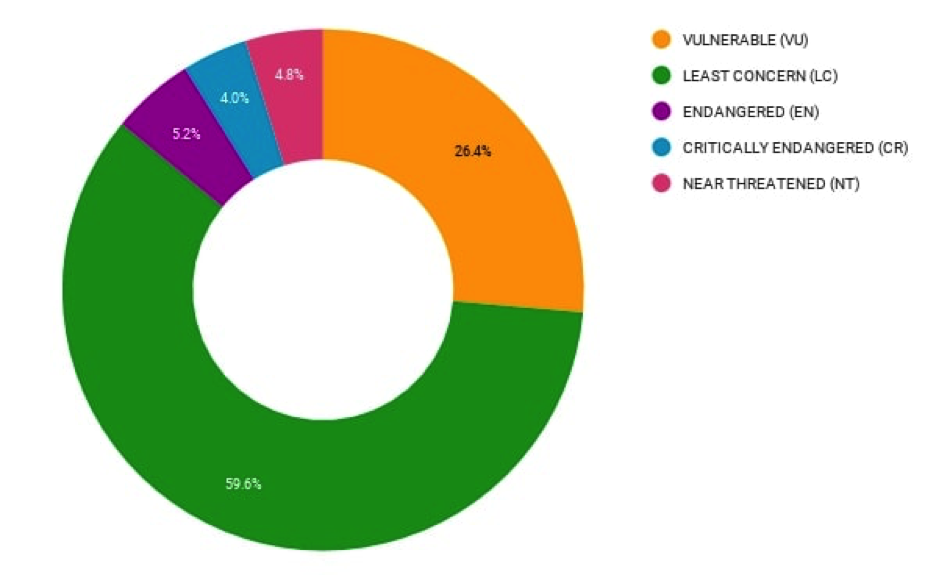

Supplement: S2 Fig — This figure shows total PNW seizures during the study period (1999–2016) by IUCN Red List status. (TIFF) [file pone.0234197.s004.tiff]
